# Trusted Computing Strengthens Cloud Authentication

**DOI:** 10.1155/2014/260187

**Published:** 2014-02-18

**Authors:** Eghbal Ghazizadeh, Mazdak Zamani, Jamalul-lail Ab Manan, Mojtaba Alizadeh

**Affiliations:** ^1^Universiti Teknologi Malaysia, 54100 Kuala Lumpur, Malaysia; ^2^MIMOS Berhad, Technology Park Malaysia, 57000 Kuala Lumpur, Malaysia

## Abstract

Cloud computing is a new generation of technology which is designed to provide the commercial necessities, solve the IT management issues, and run the appropriate applications. Another entry on the list of cloud functions which has been handled internally is Identity Access Management (IAM). Companies encounter IAM as security challenges while adopting more technologies became apparent. Trust Multi-tenancy and trusted computing based on a Trusted Platform Module (TPM) are great technologies for solving the trust and security concerns in the cloud identity environment. Single sign-on (SSO) and OpenID have been released to solve security and privacy problems for cloud identity. This paper proposes the use of trusted computing, Federated Identity Management, and OpenID Web SSO to solve identity theft in the cloud. Besides, this proposed model has been simulated in .Net environment. Security analyzing, simulation, and BLP confidential model are three ways to evaluate and analyze our proposed model.

## 1. Introduction

The new definition of cloud computing has been born by Amazon's EC2 in 2006 in the territory of information technology. Cloud computing has been revealed because of commercial necessities and makes a suitable application. According to NIST definition, “cloud computing is a model for enabling ubiquitous, convenient, on-demand network access to a shared pool of configurable computing resources.” Resource pooling, on-demand self-service, rapid elasticity, measured serviced, and broad network access are five vital features of cloud computing. Cloud has various attributes. It can be executed; supplies materials; can be organized and is in infrastructure based. These qualities provide rapid and unrestricted automatic access [1].

“The push for growth in 2011 is leading to changes in emphasis,” said M. Chiu. Also he said that IT is now recognized by business transformation, or better said that digitalization is accelerating. Cloud computing and IT management according to statistics are of top ten business and technology priorities of Asian CIOs strongly. Cloud computing, IT management, mobile technology, virtualization, business intelligence, infrastructure, business process management, data management, enterprise application, collaboration technologies, and network data communication are the top ten technology priorities. Cloud will move to become the most popular business worldwide. It shows that business and technology have been dominated by cloud computing. Therefore, thousands of new business are released every day about cloud computing technology [2].

### 1.1. Security and Cloud Computing

While cost and ease are two top benefits of cloud, trust and security are two concerns of cloud computing users. Cloud computing has claimed insurance for sensitive data such as accounting, government, and healthcare and brought opportuneness for end users. Virtualization, multitenancy, elasticity, and data owners which traditional security techniques cannot solve security problems for them are some new issues in the cloud computing. Trust is one of the most important issues in cloud computing security; indeed, two trust questions are in cloud computing. The first question is do cloud users trust cloud computing? And second question is how to make a trusted base environment for cloud users? However, when it comes to transfer their businesses to cloud, people tend to worry about the privacy and security. Is CSP trustworthy? Will the concentrated resources in the cloud be more attractive to hackers? Will the customers be locked in to a particular CSP? All these concerns constitute the main based obstacle to cloud computing. Based on these questions and also to address these concerns when considering moving critical application, cloud must deliver a sufficient and powerful security level for the cloud user in terms of new security issues in cloud computing [3].

Abbadi and Martin illustrated that trust is not a novel concept but users need to understand the subjects associated with cloud computing. Technology and business perspective are two sides of trust computing. The emerging technologies that best address these issues must be determined. They believed that trust means an act of confidence, faith, and reliance. For example, if a system gives users insufficient information, they will give less trust to the system. In contrast, if the system gives users sufficient information, they will trust the system well. There are some important issues with trust. Control of our assets is the most important security issue in cloud computing because users trust a system less when users do not have much control of it. For example, bank customers use a confidentiality ATM because when they withdraw money from an ATM machine, they trust it [4].

Cloud computing suppliers should prepare a secure territory for their consumers. A territory is a collection of computing environments connected by one or more networks that control the use of a common security policy. Regulation and standardization are other issues of trust that PGP, X509, and SAML are three examples for establishing trust by using standard. Access management, trust, identity management, single sign-on and single sign-off, audit and compliance, and configuration management are six patterns that identify basic security in cloud computing. Computer researchers believe that trust is one of the most important parts of security in cloud computing. There are three distinct ways to prove trust. First, we trust someone because we know them. Second, we trust someone because the person puts trust on us. Third, sometimes we trust someone because an organization that we trust vouches for that person. In addition, there are three distinct definitions of trust. First, trust means the capability of two special groups to define a trust connection with an authentication authority. Second, trust identifies that the authority can exchange credentials (X.509 Certificates). Third, it uses those credentials to secure messages and create signed security token (SAML). The main goal of trust is that users can access a service even though that service does not have knowledge of the users [5].

Trusted computing grows with technology development and is raised by the Trusted Computing Group. Trusted computing is the industry's response to growing security problems in the enterprise and is based on hardware root trust. From this, enterprise system, application, and network can be made safer and secure. With trusted computing, the computer or system will reliably act in definite ways and thus works in specific ways, and those performances will be obligated by hardware and software when the owner of those systems enabled these technologies. Therefore, using trust causes computer environments to be safer, less prone to viruses and malware, and thus more consistent.

In addition, trusted computing will permit computer systems to propose improved security and efficiency. The main aim of trusted computing has prepared a framework for data and network security that covers data protection, disaster recovery, encryption, authentication, layered security, identity, and access control [6].

### 1.2. Cloud Computing and Federated Identity

One of the problems with cloud computing is the management and maintenance of the user's account. Because of the many advantages of cloud such as cost and security, identity management should promise all the cloud advantages. Therefore, the new proposed model and standards should make use of all the cloud advantages and prepare an environment to ease the use of all of them.

Private cloud, public cloud, community cloud, and hybrid cloud are four essential types of cloud computing that were named by cloud users and venders. The community cloud definition shares infrastructure for definite community between organizations with common concern and private or internal cloud shares cloud services between a classified set of users. Attending to establish the best digital identity for users to use is the most important concern of cloud providers. Cloud prepares a large amount of various computing resources; consequently, diverse users in the same system can share their information and work together. Classified identity and common authentication are an essential part of cloud authentication and federated identity acts as a service based on a common authentication even though it is broadly used by most important companies like Amazon, Microsoft, Google, IBM, and Yahoo [7].

Today's internet user has about thirteen accounts that require usernames and passwords and enters about eight passwords per day. It becomes a problem and internet users face the load of managing this huge number of accounts and passwords, which leads to password tiredness; indeed the burden of human memory, password fatigue may cause users to use password management strategies that reduce the security of their secured information. Besides, the site centric Web makes online and each user account is created and managed in a distinct administrative domain so that profile management and personal content sharing will be difficult. SSO or Web single sign-on systems are invited to address the mentioned problem with the site centric. Web users, identity provider and service provider are three parts of SSO and they have a distinct role in identity scenario. An IdP collects user identity information and authenticates users, while an RP trusts on the authenticated identity to make authorization decisions. OpenID is a promising and open user centric Web SSO solution. More than one billion OpenID accounts have been permitted to use service providers according to the OpenID Foundation. Furthermore, the US Government has cooperated with the OpenID Foundation in the provision of the Open Government Initiative's pilot acceptance of OpenID technology [8].

Single username and password is an essential part of single sign-on protocols in terms of authenticate crossway numerous systems and applications, efforts to statement privacy, and security issues for web users. There are three widespread Web SSO implementations that named that OpenID, Passport/Live ID, and SAML will be enclosed along with details of common weaknesses and issues [9].

Web SSO solutions were initially advanced by numerous educational institutions in the mid-1990s. For example, Cornell University's SideCar, Stanford University's WebAuth and Yale's Central Authentication System (CAS) were entirely early reformers in the field [10].

SSO today has a critical role in cloud security and becomes essential to realize how secure the deployed SSO mechanisms truly are. Nevertheless, it is a new idea and no previous work includes a broad study on commercially deployed web SSO systems, a key to understanding of what extent these real systems are subject to security breaches [11].

In addition, Wang and Shao showed that weaknesses that do not illustrate on the protocol level could be brought in by what the system essentially agrees to each SSO party to do [12].

### 1.3. Background of the Problem

Madsen et al. in [13] defined some problems of federated identity and illustrated that FIM or Federated Identity Management established on standards permits and simplifies joining federated organizations in terms of sharing user identity attributes, simplifying authentication and allowance, or denying using service access requirements. The definition of SSO is defined as using it facility user authenticates only one time to home identity provider and logging in to access successive service providing service providers within the federated identity. There are some active problems and concerns in a federated identity environment like misuse of user identity information through SSO capability in service providers and identity providers, user's identity theft, service providers and identity providers, and trustworthiness of the user. Federated identity has identified that regardless of good architectures it still has some security problems that should be considered in the real estate implementation.

Wang in [9] discovered another problem of federated identity that is switching authentication mechanisms to an SSO solution. It means further education of the users is required and possible loss of the user base if the transition is not smoothly executed. Also worsening the situation is the lack of demand from users for a Web SSO solution. Studies have shown that users are already satisfied by their own password managers.

### 1.4. Scope and Limitation

This research focuses on trust in cloud computing. Trust in the cloud is a critical part in cloud security. As it has been mentioned in the introduction, access management, trust, identity management, single sign-on and single sign-off, audit and compliance, and configuration management are six patterns that identify basic security in cloud computing. This study will focus on federated identity which is used for user identity management. In addition, there are several federated identity architectures for managing this problem. Hub and spoke model, free form model, and hybrid model are three models for implementing federated identify in trusted computing. Although, there are some remaining issues and challenges in these management trust models so this study will focus on the hybrid model to enhance and propose architecture.

Stopping Phishing attacks completely is very difficult. Therefore, the aim of this study is to decrease the number of identity theft and Phishing attacks. Trusted computing that is applied in this study tries to decrease the success probability of Phishing attack and identity theft. However, as TPM in OpenID has been leveraged, other attacks, apart from Phishing, attempt to deal with sensitive information as an asset of users. Furthermore, in this study attacks that compromise users' computers have been ignored. Also key loggers and rootkits and cookie attacks that look like cross site request forgery (CSRF) attacks and cross site script (XSS) attacks have been ignored. Finally, attacks which compromise the integrity of the website will not be discussed.

In conclusion, this study's scope is single sign-on authentication by using some of the protocols like SAML, OAuth, and OpenID. As it has been mentioned, user uses these authentication models in many APIs uses these authentication models. Visual Studio 2012 and SQL Server 2012 have been used for simulation of the proposed model.

### 1.5. Related Work

You and Jun in [14] proposed Internet Personal Identification Number or I-PIN technique to make stronger user authentications with the cloud OpenID environment against phishing problem of relying parties which means users could obtain internet service with the OpenID, although, forcing in OpenID territory in some countries has been found by them. Besides, they analyze and evaluate their methods in comparison with the existing OpenID method, and they recommend some ways to secure OpenID environment. In their method user has to choose only 1 company out of 3 companies which delivers OpenID and the index to be a member in order to obtain OpenID service. If a user receives I-PIN information from main confirmation authority via IDP with creation OpenID, the problem of main authentication that OpenID has could be solved.

In addition, they evaluated their study and after comparison with an existing OpenID they confirmed that authentication is secure and safe against attack and private information exposition. Also they found the problem of Phishing and shown that the existing OpenID system could be solving Phishing problem and relying party authentication guarantees security against the exposition of user's passwords and ID.

Ding and Wei in [15] proposed a multilevel security framework of OpenID authentication. They claimed that their recommended framework is appropriate for all types of rule-based security model. They presented the basic OpenID infrastructure based on the single sign-on explanations that included hierarchy, OpenID components, and other security issues. Next they proposed a security access control structure for OpenID based on BLP model, which can be used to resolve the difficulty on the access control of multilevel security, and finally they store the security label in the XML document.

Mat Nor et al. in [16] took initiative remote user authentication protocol with both elements of trust and privacy by using TPM (VTPM). Ding and Wei investigated the security access control scheme for OpenID based on BLP model, which can be used to solve the problem on access control of multilevel security with storing the security label in XML document. Sun et al. [17] discussed the problem of Web SSO adoption for RPs and argued that solutions in this space must offer RPs sufficient business incentives and trustworthy identity services in order to succeed. Fazli BinMat Nor and Ab Manan [18] proposed an enhanced remote authentication protocol with hardware based attestation and pseudonym identity enhancement to mitigate MITM attacks as well as improving user identity privacy.

Latze and Ultes-Nitsche in [19] proposed using the trusted TPM (VTPM) for ensuring both an e-commerce application's integrity and binding user authentication to user credentials and the usage of specific hardware during the authentication process. Urien in [20] illustrated strong authentication (without password) for OpenID, according to a plug and play paradigm, based on SSL smart cards against Phishing attack. Khiabani et al. in [21] discovered previous trust and privacy models in pervasive systems and analyzed them to highlight the importance of trusted computing platforms in tackling their weaknesses.

### 1.6. Paper Organization

The rest of this paper is organized as follows. In the next section, we discuss the proposed solution in detail.  Section 3 provides a simulation of trusted OpenID. In Section 4, we consider issues that may arise when implementing the solution and analysis based on these issues in three approaches, and Section 5 summarizes our conclusions.

## 2. Proposed Solution

In this part, the proposed procedure will be explained. Programming languages help this study to evaluate the efficiency of the procedure. Waterfall model will be utilized to design and implement the prototype. Thus, firstly the requirements will be analyzed and secondly the algorithms based on the requirements will be proposed to solve the problem. Thirdly, the implantation steps and the essential functions are explained.

First step in designing software is requirement analysis. There are a few activities that need to be done before designing the algorithm. This research will be conducted using an experimental and modeling approach. It will begin with in-depth reading to understand the state of the art in the areas of cloud computing authentication. One of the main requirements of this study is a flow diagram of cloud authentication. Some of the best techniques that have been proposed were discussed in the related work. The advantages and disadvantages of those works were discussed. Therefore it seems that there are still open issues that are required to be searched.

**Figure 1:**
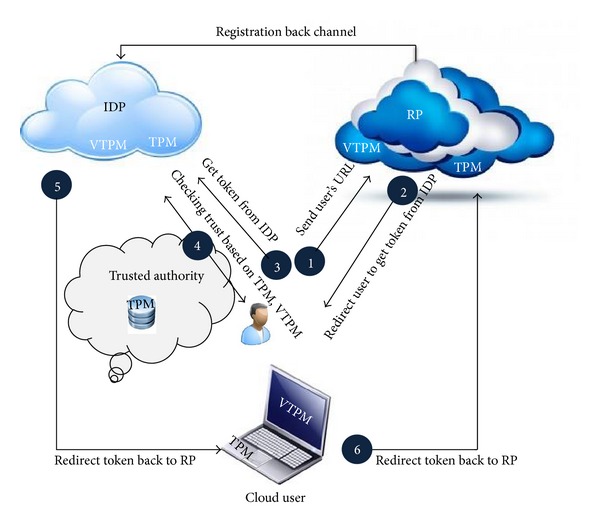
Proposed trust based federated identity architecture.

This model is based on a hybrid model architecture which is a mix of both the hub and spoke and free form model. The hybrid model will also be found in organizations that mix traditional or legacy computing with a public or private cloud environment. The aim is to make a seamless authentication environment that is based on the hybrid model. It has been trying to bring some consideration for time table between private clouds and choose the best way to achieve single sign-on. According to Figure 1, trusted authority to make a good relation between IDP and RP to prevent ID theft has been used.

This proposed model in comparison with the previous proposed models in the related work should grab authentic cloud services using the user's privacy policies, an independent third party, and ensure mutual protection of both clients and providers as main goals to afford identity management and access control. Cloud computing, SSO, and trusted computing are highlighted to support to the tasks of OpenID authentication.

To avoid a weak and vulnerable link in enterprise security, trust must be established in all the components in the infrastructure. This requires establishing a relationship, considering the identity of all of the devices and parties within the trust domain, to exchange essential key secrets that allows signing any messages that are sent back and forth. The novelty lies in the fact that the proposed model has not been offered by OpenID yet. Combining trusted computing, cloud computing, and federated identity, the model can contribute to security and privacy of cloud computing. To our knowledge, this model has not been proposed so far and we intend to pursue this model in our future work.

Binary attestation and Direct Anonymous Attestation (DAA) are two types of remote attestation. Binary attestation which is static in nature and cryptographic protocol which allows the remote authentication of a trusted platform whilst preserving the user's privacy is DAA. In our proposed model, binary attestation is used for checking integrity of users, RPs, and IDPs.

Cloud computing users, IDPs, and SPs can use a Trusted Platform Module to authenticate their hardware devices. Since each TPM (VTPM) chip or Virtual TPM (VTPM) has a unique and secret RSA key burned in it as it is produced, it is capable of performing platform authentication. On the trusted platform each system should be independent of any trusted third party. Besides Independence, each system should be able to unambiguously identify users that can be trusted both across the Web and within enterprises.

In comparison with the related work, the essential goal of the proposed model is to provide access control and identity management which are the following:

- being an independent third party;
- authenticate cloud services using the user's privacy policies, providing minimal information to the SP;
- ensure mutual protection of both clients and providers.

**Figure 2:**
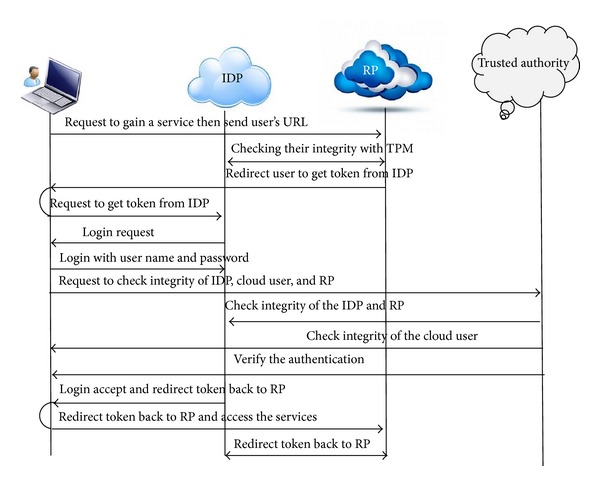
Trusted based model sequence diagram.

**Figure 3:**
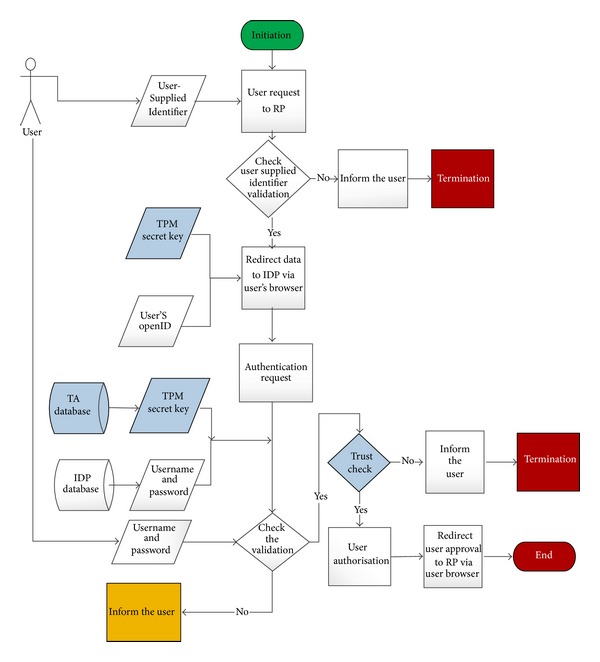
Trusted based model sequence flow chart.

Using trusted computing in OpenID should be platform independent and flexible enough to meet the challenges of today's scalable computing environments. The proposed scenario is as in Figure 1. This figure shows that Trusted Authority to handles trust relationships between user's browser, RP, and IDP. The negotiations between them show a six-step scenario. This proposed model highlights the use of the TPM (VTPM), Virtual TPM (VTPM), OpenID protocol which try to support the tasks of authentication, authorization and identity federation. Beyond these objectives, the main contribution of our work is the implementation in the cloud computing. In addition, Figure 2 shows the trusted based model sequence diagram based on trusted OpenID proposed model. Besides, Figure 3 illustrates the proposed model flowchart and highlights Trusted Authority (TA) and TPM (VTPM) roles in this model.

### 2.1. Log on Using the User's URL

OpenID allows us to sign into websites using a single identifier in the form of a URL. This URL is used instead of our username and password and it has been named Personal Identity Portal (PIP) URL. For example, in the Symantec site for user Bob, the PIP URL is bob.pip.verisignlabs.com and he can use many sites that support OpenID instead of username and password. In this example, these sites are service providers and the Symantec is identity provider. PIP helps us eliminate the need to remember different usernames and passwords for our service providers. PIP also delivers the elasticity and flexibility to share only the information we choose with each service provider. Furthermore, by creating a PIP account, we will receive a PIP URL that we can use to sign in or register at any service provider that supports OpenID and displays the OpenID logo.

### 2.2. Redirection for Client Authentication

In this step, the SP locates the user's location and creates an authentication token. SP asks the user to prove that he/she is who he/she is. For this reason, after getting the RP's request, the browser performs the next step. In the OpenID Authentication version 2.0 the IDP and RP establish an association with RSA technique secret key whereas it does not require to associate with RSA by using TPM (VTPM) hash code.

### 2.3. Client Authentication with IDP

In this scenario, the browser proceeds with token exchange based on SAML protocol. Here, users have three options to prove their identity to IDP, depending on the context of the interaction. Firstly, proving their identity, this is inserting his/her user name and password to prove his/her identity. Secondly, this can be done by using browser cookies if the users activate them. Thirdly, it can be done through using some software like SeatBelt extension in Firefox browser which is a plug-in that helps users when signing into OpenID sites, IDP using PIP URL. SeatBelt extension detects that the user has clicked on an OpenID sign-in field and prompts users to sign in. Finally, after first-time sign in, subsequent requests to SeatBelt will automatically return the user to the OpenID sign-in page with his/her PIP already filled in.

### 2.4. Mutual Attestation

Step 4 is the most critical part of our proposed OpenID trust based Federated Identity Architecture. In our environment we have assumed that IDP can collude with SP to connect user's alliance and collect user's behaviors. Also we have supposed that there is no Privacy Enhancing Technology (PET) organized in our cloud environment. Using Trusted Authority (TA) as the core, user's browser, Relying Party (RP) or Service Provider (SP), and IDP must prove their identity based on mutual attestation process using their TPM- (VTPM-) enabled platforms and verified by the TA. We have assumed that the Trusted Authority is trustworthy and must be sanctioned by the country's government or authorized by all participating parties in the Federated Identity Architecture. In this scenario TA is attester that sends the challenge to attestees (IDP, USER, and SP) to check their integrity. TA by default uses binary remote attestation to check their integrity but in this scenario uses DAA technique regarding integrity checking.

### 2.5. Authorizing User's Browser

If and only if the mutual attestation process has been successful, that is, the user and IDP have confidence in each other, then the IDP will deliver SAML token to the user's browser. Today, some of the IDPs like Google, Facebook, and IBM use SAML token and deliver their users access rights through SAML token. For future work we may consider other mechanisms.

### 2.6. User's Browser Request

In this step, the user puts more confidence in the SP for getting a service base on the SAML token. IDP sends a encrypt token by the user's public key that shows IDP is legitimated and verified by a trusted authority. User decrypts the token by his/her private key. Finally, the user uses the token to get more services from RP or SP.

It is important to note that the TA in our proposed architecture will need to deal with Virtual Machine (VM). It will be associated with different services requested by the client. Hence, TA needs to attest each virtual TPM (VTPM) that is associated with each VM on Client Machine. Finally, this paper highlights the use of an OpenID, trusted computing, and federated identity which deliver provision to make secure authentication, authorization, and identity federation. Trusted computing is very flexible with regards to its use in a cloud environment allowing a service to be provided securely and reliably. In comparison with other frameworks in related work, our proposed model has more strength because of better security management, shifted risk management, private remote attestation, and efficient binding of identity assertions.

## 3. Simulation of Trusted OpenID

As mentioned before the overall flow of OpenID authorization works are signed up for an OpenID Consumer Key, perform discovery, get a pre-approved request token, and continue with the OpenID Flow. In addition to this flow, in order to evaluate the process we could find the vulnerabilities of OpenID flow to better understand the behavior of the proposed model in the real world.

In this section we turn our attention to simulate the purpose algorithm and design to develop this model based on simulation in C#. Our system, referred to as Trust OpenID, is composed of different component which has its own tasks. For now, based on our scope we run our system on the virtualization system but in the future work we will explain that our system has this ability to be located in the cloud environment and integrated with VMware, Microsoft Azur, XenServer. Our system will observe the data flow of OpenID, request, discover, approve and authorize the user and providers. In this simulation first we fetch all the vendors, process, request, discovery between the user's browser and service and identity providers. On the other hand we are observing and monitoring data exchange between objects and subjects.

**Figure 4:**
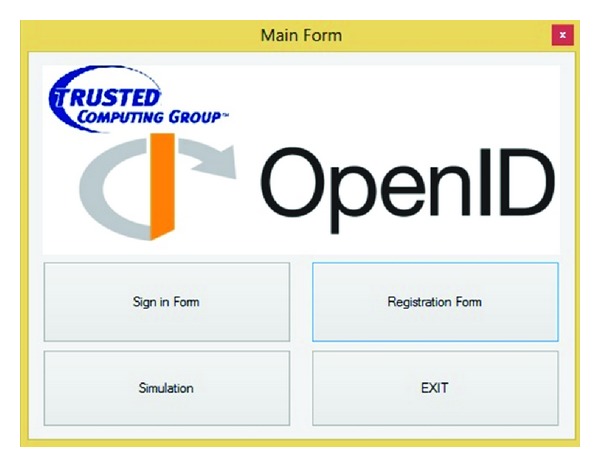
Main form of simulation.

As it is illustrated in Figure 4, the main page contains sign-in form, registration form, simulation, and exit. This simulation should cover the communication type, generating signature, initiation and discovery, establishing association, request authentication, responding to authentication request, and verifying assertion. The first step in using OpenID is to set up an account with one of the many providers on the web. Registration form helps us to register just one time for getting and using OpenID account. Next in sign-in form after getting OpenID account, user can use her or his OpenID account to get the service's SP by IDP account.

If the user is thinking that OpenID sounds a lot like Facebook Connect, he or she can use this credential to use other services of other sites which accept Facebook Connect. It allows users to sign into websites using their Facebook credentials.

**Figure 5:**
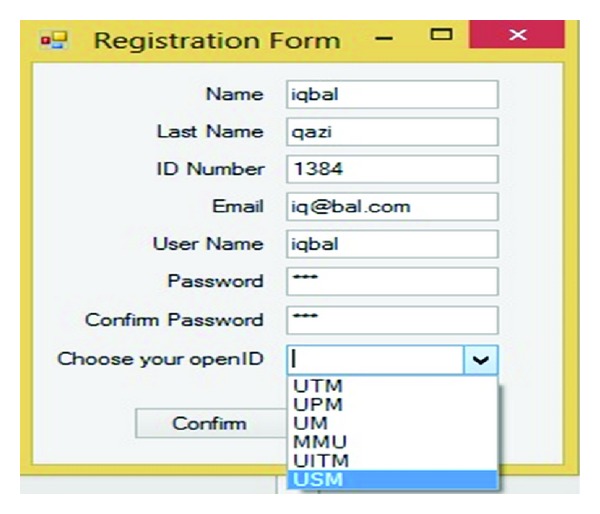
Registration Form.

**Figure 6:**
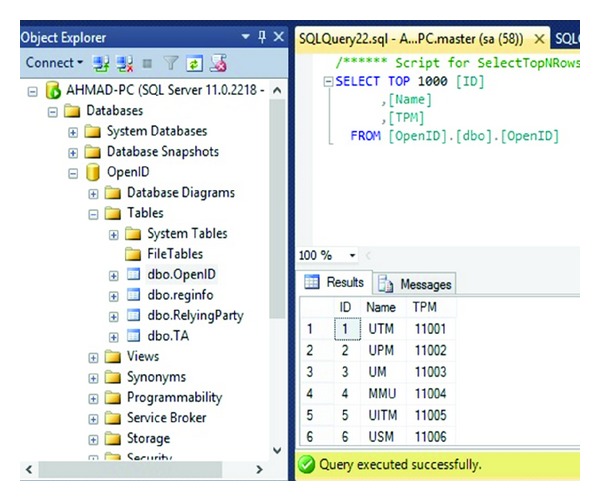
IDP database.

In order for an OpenID Connect Client to utilize OpenID services for a user, the Client needs to register with the OpenID Provider to acquire a Client ID and shared secret.  Figure 5 describes how a new Client can register with the provider, and how a Client already in possession of a Client ID can retrieve updated registration information. The Client Registration Endpoint may be coresident with the token endpoint as an optimization in some deployments. This study assumes that UTM, UPM, UM, MMU, UITM, and USM are identity providers.  Figure 6 shows IDP's database which uses dbo. OpenID to save data.

**Figure 7:**
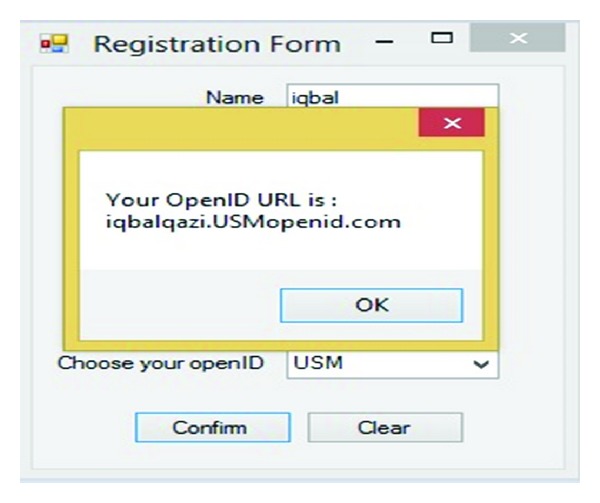
Confirmation of registration.

**Figure 8:**
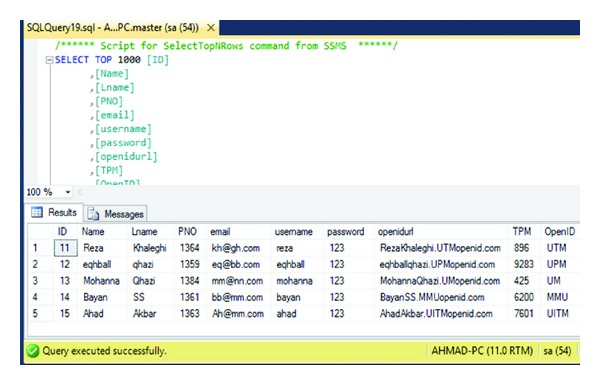
Registration database.

The user sends request encoded as a post with the Name, Last Name, ID Number, Email, username, Password, and Identity provider parameters added to the HTTP request to the IDP. Also Figure 6 illustrates the registration information database which is containing registration information and TPM (VTPM) and OpenID URL of the users.  Figure 7 shows confirmation of the registration part and OpenID URL which has been issued by IDP. In this step with OpenID acceptance, the TPM (VTPM) also saves in the dbo.reginfo SQL database as shown in Figure 8. Also it shows the OpenID URL which assigned by the IDP provider.

### 3.1. Log on Using the User's URL

**Figure 9:**
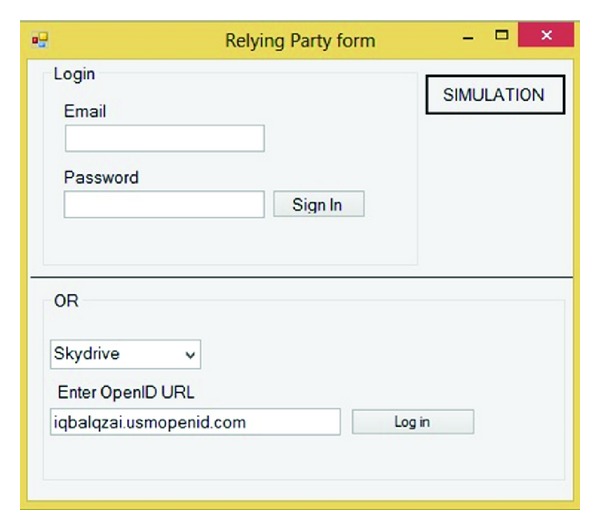
Simulation of RP website.

Based on the proposed model as mentioned before, the first step is logged on in service provider by OpenID URL. OpenID allows us to sign into websites using a single identifier in the form of a URL. This study assumed that service providers are Skydrive, Dropbox, and MSN which accepted our IDPs. First in RP combo user choose the RP and after that user should enter the OpenID URL or PIP.  Figure 9 simulates that user assigned to Skydrive by iqbalqazi.usmopenid.com.

### 3.2. Redirection for Client Authentication

**Figure 10:**
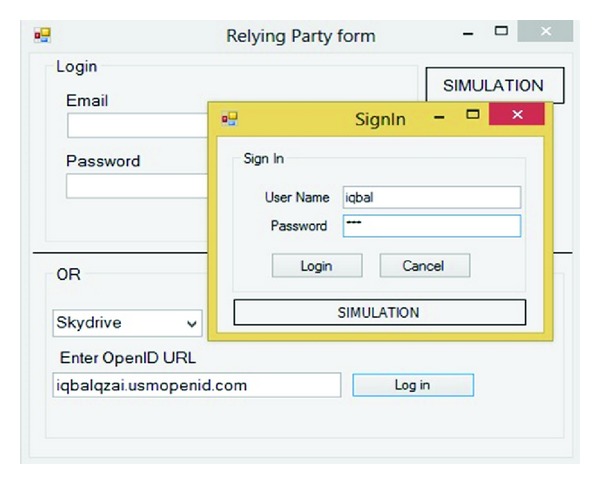
IDP Sign on.

In this step, the SP redirects user to IDP to prove his or her identity and get appropriate token. For this reason, after getting the RP's request, sign-in form will appear.  Figure 10 shows this step.

### 3.3. Client Authentication with IDP

In this step based on Figure 10, the user should prove his or her identity. As mentioned before the user has three options to prove identity to IDP, depending on the context of the interaction. In this simulation user is asked to enter username and password.

### 3.4. Mutual Attestation

**Figure 11:**
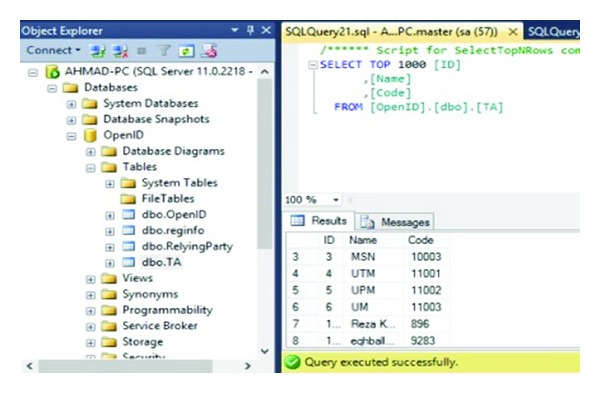
TA database.

Checking user's integrity is the aim of this step. First of all IDP checks the username and password based on dbo.reginfo. Next, TA checks the integrity based on the TPM (VTPM). In this study it is assumed that TA after attestation has all the TPM (VTPM) in its database. Code in dbo. TA means TPM (VTPM) of the all vendors and users which has been shown in Figure 11.

**Figure 12:**
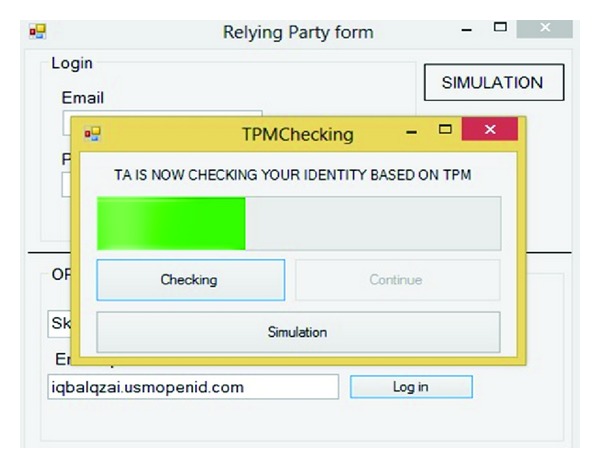
TPM checking.

**Figure 13:**
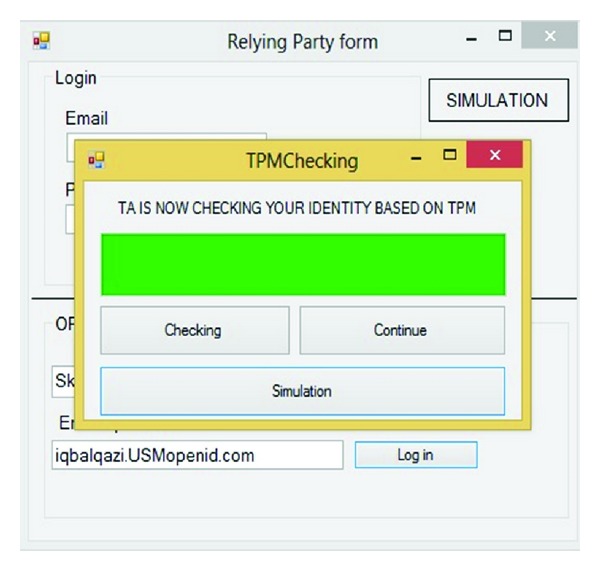
Pass TPM checking.

This step is the most important part and has been emphasized by hackers. Simulation after checking username and password checks the TPM (VTPM) after clicking on the Check button. If the result of checking the username and password was correct the continue button will be highlighted otherwise the Phishing page will appear. Figures 12 and 13 show this process.

### 3.5. Authorizing User's Browser

**Figure 14:**
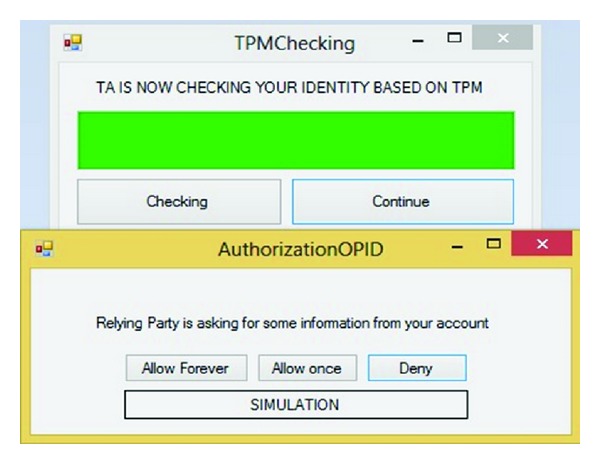
User authorization.

If and only if the previous process has been successful which means the user and IDP have confidence in each other. Based on OpenID privacy user can determine how long the SP accepts her or his approved identity.  Figure 14 illustrates that user can choose Allow forever, Allow once, and Deny. IDP delivers SAML token to RP via user's browser.

### 3.6. User's Browser Request

**Figure 15:**
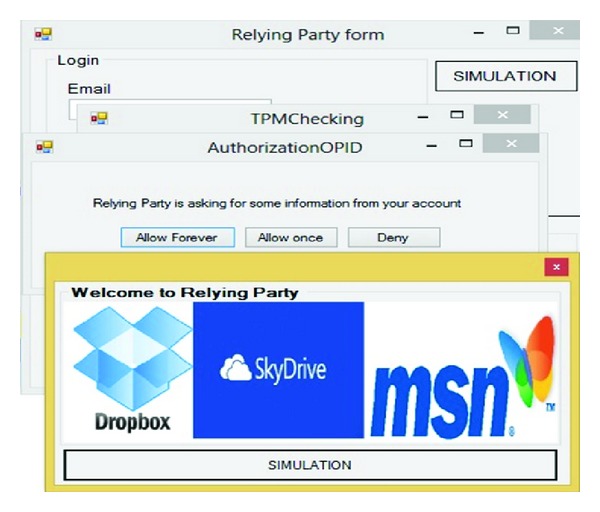
RP accepts user's credential.

Figure 15 illustrates the confidence between the user and RP; that is, user could get a service based on the SAML token.

In this part we explained trusted based model, sequence and flow chart to mitigate identity theft in OpenID single sign on environment. Next part shows the evaluation and test of this proposed model.

## 4. Test and Evaluation

In this part, the simulation and design of the proposed solution will be discussed in detail. Objectives, subjects, and their attributes are three considerations of each system. The details of the subjects and objects were discussed and then the process was explained; finally the Trusted OpenID will be analyzed by three-approache sample tests. Design phase, architecture, flowchart, and flow diagram of the proposed model have been shown and discussed in Section 3. Then analyzing the results of the proposed design must fulfill the objectives of the project which is using trusted computing against identity theft in a cloud identity environment especially in OpenID authentication. Confidentiality analysis, simulation analysis, and security analysis are three methods to evaluate and test the proposed model.

### 4.1. Confidentiality Analysis

Key role in establishing computer system is the security model which has been used to express the security policy. Availability, integrity, and confidentiality are three most important concerns in security area where confidentiality is the aim of this research and in this part we will explain how our proposed try to confidence authentication in a cloud environment by using Trusted computing. There are some security models which attend to gain security in their enterprises. For example, Biba and Bell&LaPadula (BLP) are two most popular security models; Biba focused on integrity in recognized mathematical terms and BLP is the most classic confidentiality model focused on confidentiality. In this study BPL will be used as a basic theory for confidentiality prove in multilevel security Trusted OpenID and as a way for evaluating the proposed model, it has been focused on confidentiality of trusted OpenID mechanism. Also, in case of adopting the BLP model to OpenID we focused on the subjects, objects, and their data exchange of users' application to enhance the confidentiality and security of Trusted OpenID mechanism besides bring the user convenience.

#### 4.1.1. BLP Model

Military affair is a good example for using BLP model all the same the designer of computer security utilized and applied to contribute to computer security. While this model has so many advantages, but there are some disadvantages such as forgetting access history [15]. Security level and access matrix are two main parts in BLP model which define the access permissions. Security policies accept information flowing upwards from a low security level to a high security level and also prevent information flowing downwards from a high security level to a low security level.

#### 4.1.2. Terminology

*Identifier (I):* an Identifier is either an “http” or “https” URI, commonly referred to as a “URL” within this document or an XRI (XRI_Syntax_2.0). This document defines various kinds of Identifiers, designed for use in different contexts.

*User-Agent (UA):* the end user's Web browser which implements HTTP/1.1. User-Agents will primarily be common Web browsers

*Relying Party (RP):* a Web application that wants proof that the end user controls an Identifier. 

*OpenID Provider (IDP):* an OpenID Authentication server on which a Relying Party relies for an assertion that the end user controls an Identifier.

*IDP Endpoint URL (IEU):* the URL which accepts OpenID Authentication protocol messages, obtained by performing discovery on the User-Supplied Identifier. This value must be an absolute HTTP or HTTPS URL. 

*IDP Identifier (II):* an Identifier for an OpenID Provider. 

*User-Supplied Identifier (USI):* an Identifier that was presented by the end user to the Relying Party, or selected by the user at the OpenID Provider. During the initiation phase of the protocol, an end user may enter either their own Identifier or an IDP Identifier. If an IDP Identifier is used, the IDP may then assist the end user in selecting an Identifier to share with the Relying Party. 

*Claimed Identifier (CI):* an Identifier that the end user claims to own; the overall aim of the protocol is verifying this claim. 

*IDP-Local Identifier (ILI):* an alternate Identifier for an end user that is local to a particular IDP and thus not necessarily under the end user's control. http://eqhball.pip.verisignlabs.com.

*Generate (G):* an identifier which has been generated by TPM or VTPM.

*Challenge (CH):* TA is attester that sends the challenge to attestees (IDP, USER, and RP) to check their integrity. As mentioned before TA by default uses binary remote attestation to check their integrity but in this scenario uses DAA technique regarding integrity checking. 

*Trusted Authority (TA):* an enterprise to verify mutual attestation process using the TPM- (VTPM-)enabled platforms.

**Figure 16:**
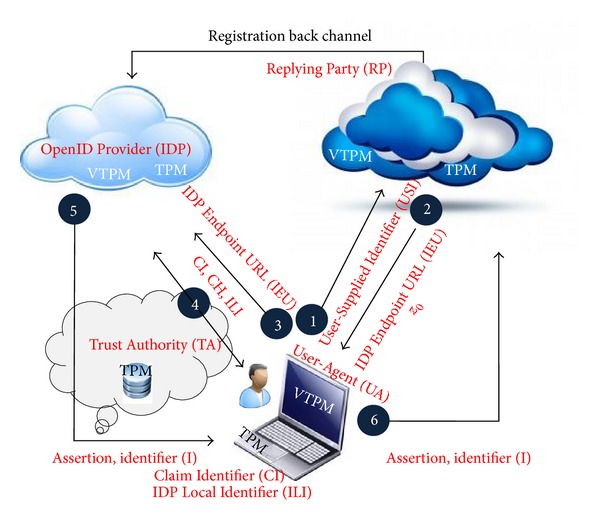
Objects and subjects of the proposed model.

Figure 16 shows completely the objects and subjects of the proposed model and the place based on the final OpenID authentication architecture. These subjects and objects help to end user to prove that he or she owns a URL or XRI through a series of communications between the user and the website to which he/she is authenticated. The user submits the URI/XRI identifier at OpenID Relying Party through User-Agent's browser.

#### 4.1.3. Protocol Overview

Based on the OpenID authentication [22] in the first step, the end user initiates authentication by presenting a USI to the RP via their UA. To initiate OpenID Authentication, the RP should present the end user with a form that has a field for entering a USI.

Next, after normalizing the USI, RP performs discovery on USI and establishes the IEU that the end user uses for authentication. Upon successful completion of discovery, RP will have IEU and Protocol Version but if the end user did not enter II the CI and ILI will be present. In this scenario it has been assumed that the user entered the II and therefore CI and ILI will be omitted.

The RP and the IDP establish an association based on their TPM (VTPM). The IDP uses an association to sign subsequent messages and the RP to verify those messages whereas OpenID Authentication supports two signature algorithms which are HMAC-SHA256 with 256 bit key length and HMAC-SHA1 with 160 bit key length.

Once the RP has successfully performed discovery and created an association with the discovered IDU, it can send an authentication request to the IDP to obtain an assertion. An authentication request is an indirect request. In step three, RP redirects the end user's UA to the IDP with an OpenID Authentication request.

This model is indirect communication in which messages are passed through the User-Agent. This can be initiated by either the Relying Party or the IDP. Indirect communication is used for authentication requests and authentication responses. There are two methods for indirect communication: HTTP redirections and HTML form submission

When an authentication request comes from the UA via indirect communication, the IDP should determine that an authorized end user wishes to complete the authentication. If an authorized end user wishes to complete the authentication, the IDP should send a positive assertion to the RP.

Next in step four, the IDP establishes whether the end user is authorized to perform OpenID Authentication and wishes to do so.

In step five, the TA checks the trust's objectives based on their TPM (VTPM). IDP redirects the end user's UA back to the RP with either an assertion that authentication is approved or a message that authentication failed. This identifier includes openid.ns, openid.mode, openid.op_endpoint, openid.claimed_id, openid.identity, openid.return_to, openid.response_nonce, openid.invalidate_handle, openid.assoc_handle, openid.signed, and openid.sig.

If the IDP is unable to identify the end user or the end user does not or cannot approve the authentication request, the IDP should send a negative assertion to the RP as an indirect response.

Step six and the last step which the RP verifies the information received from the IDP including checking the Return URL, verifying the discovered information, checking the nonce, and verifying the signature by using the TPM (VTPM) established during the association to the IDP.

#### 4.1.4. Phishing Attack

A special type of Phishing attack is where the RP is a rogue party acting as a fake RP. The RP would perform discovery on the EUI and instead of redirecting the UA to the IDP, it would proxy the IDP through itself. This would thus allow the RP to capture credentials that the end user provides to the IDP. While TA prevents this sort of attack by checking the integrity of the RP and IDP.

#### 4.1.5. Definition of System State

**Figure 17:**
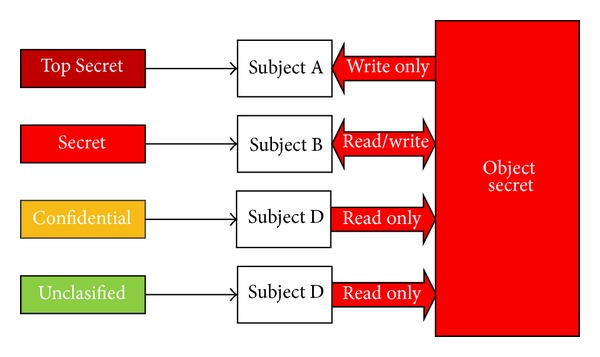
Access class matrix and relationship between objects and subjects.

**Figure 18:**
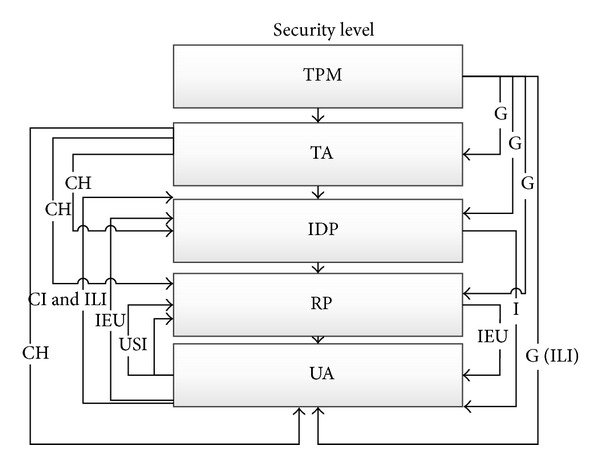
Security level, Subject, and object of the proposed model.

Our proposed model is a state machine model, and the state is expressed by Figures 16, 17, and 18. It consists of subjects, objects, access attribute, access matrix, subject functions, and object functions. It has been defined that the subjects of the proposed model are Challenge (CH), Generate TPM (VTPM) Identifier (G), User-Supplied Identifier (USI), IDP Identifier (II), IDP Endpoint URL (IEU), Identifier (I), Claim identifier (CI), and IDP Local Identifier (ILI). Also objects of the proposed model are Relying Party (RP), OpenID Provider (IDP), User-Agent (UA), Trust Authority (TA), Trusted Platform Module (TPM), and Virtual TPM (vTPM). Access attributes are Read, Write, Read and write, and execute. Access Matrix is access matrix, where each member represents the access authority of subject to object.

The proposed model state machine is *T* where each member of *T* is *t*.

1. *t* ∈ *T* where *T* is sorted quaternion.
2. *T* = (*a*, *B*, *c*, *D*), where,
3. *a*⊆(*S* × *O* × *A*),
4. *S* is a set of subjects.
5. *O* is a set of objects.
6. *A* = [*r*, *w*, *a*, *e*] is the set of access attribute.
7. *B* is access matrix, where *B*_*ij*_⊆*A* signifies the access authority of *s*_*i*_ to *o*_*i*_.
8. *c* ∈ *C* is the access class function, denoted as *c* = (*c*_*s*_, *c*_*o*_).
9. *c*_*s*_ is the security level of the subject and includes the confidentiality level *c*_1_(*S*) and category level *c*_4_(*S*).  Figure 17 shows the security level in BLP and relation between subjects and objects.
10. *c*_*o*_ signifies the security function of objects.  Figure 18 shows the relation the entire subjects, objects, security functions, security level of the proposed model. As shown in this figure confidentiality of the TPM (VTPM) is highest in the state machine and the lowest is the user agent. Therefore,
11. Confidentiality level is *c*_1_(*TPM*), *c*_2_(*TA*), *c*_3_(*IDP*), *c*_4_(*RP*), and level *c*_5_(*UA*).
12. *D* signifies the existing Hierarchy on the proposed model as shown in Figure 18.
13. *e*: *R* × *T* → *I* × *T* shows all therolese in the proposed model in which *e* is the system response and the next state. *R* is the requests set and *I* is the arbitrate set of the request which is yes, no, error, and question. In this study question is important because if the response equal to question, it means that the current rule cannot transact with this request.
14. *ω* = [*e*_1_, *e*_2_,…, *e*_*s*_], where *ω* is the list exchange data between objects.
15. *W*(*ω*)⊆*R* × *I* × *T* × *T*.
16. (*R*_*k*_, *I*_*m*_, T*, *T*) ∈ *W*(*w*).
17. If and only if *I*_*m*_≠ question and exit a unique *J*, 1 ≤ *j* ≤ *s*, it means that the current rule is valid and subject and object also are valid, because object verifies the TPM (VTPM) of the other object (attestee) by the request (challenge) for integrity checking.
18. Consequently, the result is (*I*_*m*_, t*) = *e*_*i*_(*R*_*k*_, *t*), which shows that for all the requests in the *t* there is unique response which is valid.

We should prove that each state of the proposed model is secure. It has been assumed that each state is secure except state 4 as shown in Figure 1. Therefore, if state 4 is secure all the states will be secure and based on the Section 4.1.4 this step also is secure to gain the secure state.

(19) Σ(*R*, *I*, *W*, *z*_0_) ⊂ *X* × *Y* × *Z*.
(20) (*x*, *y*, *z*) ∈ Σ(*R*, *I*, *W*, *z*_0_).
(21) If and only if (*z*_*t*_, *y*_*t*_, *z*_*t*_, *z*_*t*−1_) ∈ *W* for each *t* ∈ *T*, where *z*_0_ is the initial state. Based on the above definition, Σ(*R*, *I*, *W*, *z*_0_) is secure iff all states of the system, for example, (*z*_0_, *z*_1_, …, *z*_*n*_), are secure states.

BLP model has several axioms (properties) which can be used to limit and restrict the state transformation. If the arbitrary state of the system is secure, then the system is secure. In this study the simple-security property will be adopted.

#### 4.1.6. Simple-Security Property (SSP)

(22) *t* = (*a*, *B*, *c*, *D*)
(23) Satisfies SSP if and only if,
(24) For all *s* ∈ *S*,
(25) *s* ∈ *S*⇒[(*o* ∈ *a*(*s* : *r*, *w*))⇒(*c*_*s*_(*s*), >*c*_*o*_(*o*))],
(26) that is, *c*_1_(*s*) ≥ *c*_2_(*o*), *c*_3_(*s*)⊇*c*_4_(*o*).
(27) *c*_1_(*G*) ≥ *c*_2_(TPM),
(28) *c*_1_(IEU) ≥ *c*_2_(RP),

Based on the figure 18 and the SSP, all the objects of the proposed model which has been categorized in the proposed security level can write in the lower state. As well as all the objects can read at a higher level, but cannot read at a lower state.

Star property, discretionary security, and compatibility property are other models which can be used to limit and restrict the state transformation and they will be used in future work.

### 4.2. Simulation Environment and Evaluation

In software development lifecycle, testing and evaluation is the final step in developing the software. In fact, testing process verifies that whether the system has achieved its aim, objectives, and determined scope. Therefore, proposed model will be evaluated to show whether it could gain the mentioned objective. To do this we need to conduct a test.

**Figure 19:**
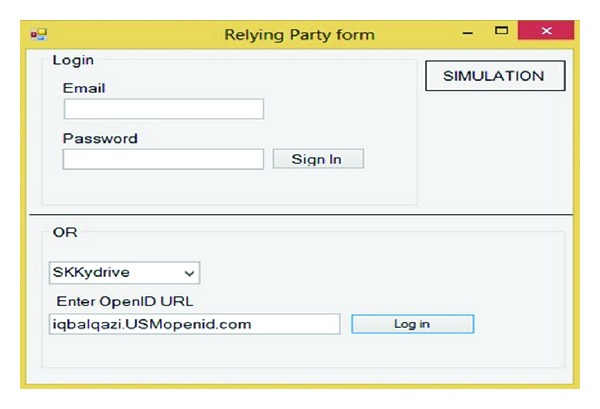
Phishing Relying Party website.

**Figure 20:**
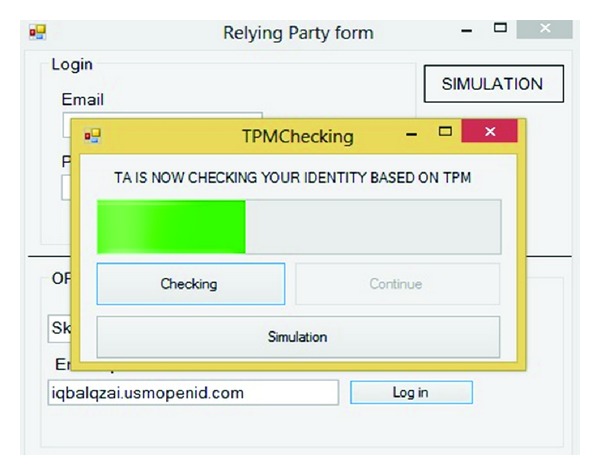
Checking identity based on TPM.

**Figure 21:**
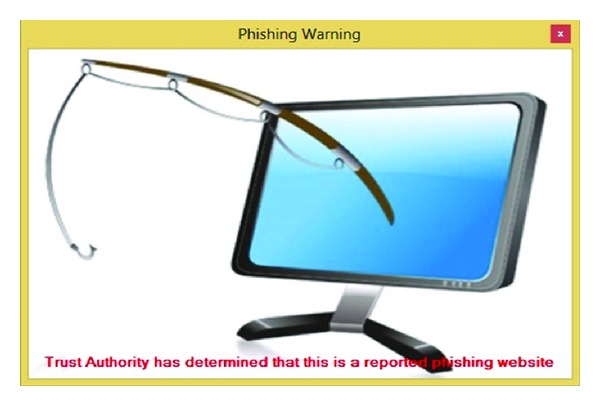
TA reports phishing message.

Based on the objectives the aim of this study is to mitigate the identity theft and Phishing attack. Thus wrong RP will be inputted to the system and then it will analyze the dataset based on the username and TPM (VTPM) conditions that have been discussed in Section 3. Figures 19, 20, and 21 show this evaluation. First, user goes in the fake service provider which is http://www.skkydrive.com/. Next, after passing username checking the TPM (VTPM) will be checked. After checking Skkydrive's TPM (VTPM) by TA, TA sends a Phishing message via user browser and reports false integrity of the fake RP.

### 4.3. Security Analysis

In this section, we examine the strengths and weaknesses of the proposed solution in terms of security and consider possible improvements.

#### 4.3.1. Mutual Authentication

Mutual authentication, also called two-way authentication, is a process or technology in which both entities in a communications link authenticate each other. In our proposed model, IDP authenticates a User-Agent by asking him/her to input the username and password. As the TA by using TPM (VTPM) approved the IDP which is only known to the user, other users cannot guess the correct TPM (VTPM) because of the TPM (VTPM) characteristics. Meanwhile, the user authenticates the RP website and deals with it presenting a USI to the RP. We assume that an attacker can send the UA to the fake IDP; thus, TA reports to the end user that he/she is on the Phishing website. The absence of a TA will cause authentication of the OpenID to fail.

#### 4.3.2. Compromising the TA

The strength of the proposed model lies in TPM (vTPM) which is a secure vault for certificates and the fact that it is difficult to compromise the components. The attacker is assumed to call TPM (vTPM) commands without bounds and without knowing the TPM (vTPM) root key, expecting to obtain or replace the user key. The analysis goal in TPM (vTPM) study is to guarantee the corresponding property of IDP, the user, RP execution, and the integrity of them [21, 23]. Also, the overall aim of the proposed model is verifying the TA. Therefore, to compromise the solution, an attacker must at least know TPM content and then target the components accordingly.

An essential axiom is TPM (vTPM) and is bound to one and only one Platform which has been used in this study to check the integrity. Hence in [24] Black Hat showed how one TPM could be physically compromised to gain access to the secrets stored inside. But Microsoft believed that using a TPM is still an effective means to help protect sensitive information and accordingly take advantage of a TPM. The attack shown requires physical possession of the PC and requires someone with specialized equipment, intimate knowledge of semiconductor design, and advanced skills.

While this attack is certainly interesting, these methods are difficult to duplicate, and as such, pose a very low risk in our proposed model. Furthermore, IDP asks the user for his/her credential to gain security assurance. As a result, an attacker must not only be able to retrieve the appropriate secret from TPM (vTPM), but also find the user credential (username and password). If the credential is sufficiently complex, this poses a hard, if not infeasible, problem to solve in order to obtain the required key to Phishing attack in OpenID environment.

#### 4.3.3. Authentication in an Untrustworthy Environment

Sometimes users have to sign into their web accounts in an untrustworthy environment, for example, accessing a credit card account using a public internet in university, shared computer, or pervasive environment. Our solution is also applicable to such cases.

In this case, the user must sign into his/her OpenID account via the trusted device, and then just follow the OpenID login instructions. In our proposed model required checking the TPM for each authentication regardless of cookies attesting the integrity of the trusted device. Because of the using TPM and trusted device, the user can read the authentication information from the device and then log into the website on the untrustworthy device.

Khiabani et al. [21] described that pervasive systems are weaving themselves in our daily life, making it possible to collect user information invisibly, in an unobtrusive manner by even unknown parties. So OpenID as a security activity would be a major issue in these environments. The huge number of interactions between users and pervasive devices necessitates a comprehensive trust model which unifies different trust factors like context, recommendation, and history to calculate the trust level of each party precisely. Trusted computing enables effective solutions to verify the trustworthiness of computing platforms in untrustworthiness environment.

#### 4.3.4. Insider Attack

Weak client's password or server secret key stored in server side is vulnerable to any insider who has access to the server. Thus, in the event that this information is exposed, the insider is able to impersonate either party. The strength of our proposed protocol is that TPM key is the essential part in the Trust OpenID protocol and TA stores TPM database with its TPM key which cannot be accessed by attackers. Therefore, our scheme can prevent the insider from stealing sensitive authentication information.

#### 4.3.5. The Man in the Middle Attack

In the man in the middle (MITM) attack, a malicious user located between two communication devices can monitor or record all messages exchanged between the two devices. Suppose an attacker tries to launch an MITM attack on a user and a website, and that the attacker can monitor all messages sent to or received by the user.

The idea of making conventional Phishing, pharming, and MITM attacks useless will apply to private users, which usually are not connected to a well-configured network. Furthermore private users often administrate their computers by themselves. Using public key infrastructures (PKI), stronger mutual authentication such as secret keys and password, latency examination, second channel verification, and one time password are some of the ways which have been released to prevent MITM in the network area.

Mat Nor et al. in [16] described that many security measures have been implemented to prevent MITM attacks such as Secure Sockets Layer (SSL) or Transport Layer Security (TLS) protocol; adversaries have come out with a new variant of MITM attack which is known as the Man-in-the-Browser (MitB) attack which tries to manipulate the information between a user and a browser and is much harder to detect due to its nature of attacks.

Trust relationship between interacting platforms has become a major element in increasing the user confidence when dealing with Internet transactions especially in online banking and electronic commerce. Therefore, in our proposed model in order to ensure the validity of the integrity measurement from the genuine TPM (vTPM), the Attestation Identity Key (AIK) is used to sign the integrity measurement. AIK is an asymmetric key and is derived from the unique Endorsement Key (EK) certified by its manufacturer which can identify the TPM identity and represent the Certificate Authority role against MITM attack.

## 5. Conclusion

In this research first we did a thorough literature review to be familiar with cloud computing, cloud architecture, federated identity, SAML, OAuth, OpenID, Trusted computing, and problems of authentication in cloud computing. Next, it has been defined that Phishing attack, identity theft, MITM attack, DNS attack are common attacks and vulnerabilities in the cloud. The related work emphasized on those researches that found any way against identity theft and Phishing attack in the federated identity environment.

The proposed algorithm that combined trusted computing with OpenID and adopted trust in federated area was explained. TPM (vTPM) is an essential part of trusted computing and also in this research; TPM (vTPM) are the main part against identity theft. The proposed model has six steps based on OpenID exchange data flow. For implementation, Visual Studio and SQL are good tools to simulate the proposed model. The last part of each project tested and evaluated the project with real world datasets. In this part, based on the statistics and security model, we proposed BLP and adopted it for the Trusted OpenID model. The main goal of this research was to gain some predefined security objectives based on the problem statement. Besides, the simulation method has been evaluated against Phishing attack and also has evaluated the strengths and weaknesses of the proposed solution in terms of security and considered possible improvements.

## References

[1] Mell P, Grance T (2011). The NIST definition of cloud computing (draft). *NIST Special Publication*.

[2] Earls A Gartner takes on cloud identity management. http://searchsoa.techtarget.com/tip/Gartner-takes-on-cloud-identity-management.

[3] Rodriguez UF, Laurent-Maknavicius M, Incera-Dieguez J (2006). *Federated Identity Architectures*.

[4] Abbadi IM, Martin A (2011). Trust in the cloud. *Information Security Technical Report*.

[5] Carmignani A (2010). *Identity Federation Using SAML and WebSphere Software*.

[6] TCG Trusted computing. http://www.trustedcomputinggroup.org/trusted_computing.

[7] Yan L, Rong C, Zhao G (2009). Strengthen cloud computing security with federal identity management using hierarchical identity-based cryptography. *Cloud Computing*.

[8] Sun S-T, Pospisil E, Muslukhov I, Dindar N, Hawkey K, Beznosov K What makes users refuse web single sign-on?: an empirical investigation of OpenID.

[9] Wang S (2011). *An Analysis of Web Single Sign-on*.

[10] Hodges H, Johansson, Morgan (2008). *Towards Kerberizing Web Identity and Services*.

[11] Armando A, Carbone R, Compagna L, Cuellar J, Tobarra L Formal analysis of SAML 2.0 web browser single sign-on: breaking the SAML-based single sign-on for google apps.

[12] Wang K, Shao Q Analysis of cloud computing and information security.

[13] Madsen P, Koga Y, Takahashi K Federated identity management for protecting users from ID theft.

[14] You JH, Jun MS A mechanism to prevent RP phishing in OpenID system.

[15] Ding X, Wei J A scheme for confidentiality protection of OpenID authentication mechanism.

[16] Mat Nor FB, Abd Jalil K, Ab Manan J-L (2011). Remote user authentication scheme with hardware-based attestation. *Communications in Computer and Information Science*.

[17] Sun S-T, Boshmaf Y, Hawkey K, Beznosov K A billion keys, but few locks: the crisis of web single sign-on.

[18] Fazli Bin Mat Nor KAJ, Ab Manan Jl (2012). Mitigating man-in-the-browser attacks with Hardware-based authentication scheme. *International Journal of Cyber-Security and Digital Forensics*.

[19] Latze C, Ultes-Nitsche U Stronger authentication in e-commerce: how to protect even Naïve user against Phishing, pharming, and MITM attacks.

[20] Urien P An OpenID provider based on SSL smart cards.

[21] Khiabani H, Manan J-LA, Sidek ZM A study of trust & privacy models in pervasive computing approach to trusted computing platforms.

[22] Ferg B (2007). *OpenID Authentication 2. 0-Final*.

[23] Donovan M, Visnyak E Seeding the cloud with trust: real world trusted multi-tenancy use cases emerge. http://www.ittoday.info/Articles/Trust/Trust.htm.

[24] Cooke P Black Hat TPM Hack and BitLocker. http://blogs.windows.com/windows/b/windowssecurity/archive/2010/02/10/black-hat-tpm-hack-and-bitlocker.aspx.

